# Integrated Transcriptome and Microbiota Reveal the Regulatory Effect of 25-Hydroxyvitamin D Supplementation in Antler Growth of Sika Deer

**DOI:** 10.3390/ani12243497

**Published:** 2022-12-11

**Authors:** Huazhe Si, Songze Li, Weixiao Nan, Jianan Sang, Chao Xu, Zhipeng Li

**Affiliations:** 1 College of Animal Science and Technology, Jilin Agricultural University, Changchun 130118, China; 2 Department of Special Animal Nutrition and Feed Science, Institute of Special Animal and Plant Sciences, Chinese Academy of Agricultural Sciences, Changchun 130112, China; 3 Jilin Provincial Engineering Research Center for Efficient Breeding and Product Development of Sika Deer, Jilin Agricultural University, Changchun 130118, China; 4 Key Laboratory of Animal Production, Product Quality and Security, Ministry of Education, Jilin Agricultural University, Changchun 130118, China

**Keywords:** 25-hydroxyvitamin D, sika deer, antler transcriptome, immune response, lipid metabolism

## Abstract

**Simple Summary:**

The antler is an important product of deer, which involves in the fast growth of bone. Previous study demonstrated that the production efficiency of 1,25(OH)_2_D is associated with the growth of antler. 25(OH)D is a 1,25(OH)_2_D precursor that is closely associated with bone development in mammals. However, the direct effects on antler growth and transcription, and the responses of the gut microbiota and metabolism of 25(OH)D supplementation in deer remain unclear. In this study, we demonstrated that 25(OH)D supplementation directly increased the growth and affected the gene expression profiles of sika deer antler. The bacteria community composition and metabolites in rumen were also changed following 25(OH)D supplementation, suggesting the enhanced metabolism of branch-amino acids. Our results confirmed the positive effect of 25(OH)D on antler growth and revealed the regulation of 25(OH)D supplementation on the host and microbes in sika deer.

**Abstract:**

The level of plasma 25-hydroxyvitamin D (25(OH)D) is associated with the growth of the antler, a fast-growing bone organ of Cervidae. However, the benefits of 25(OH)D supplementation on antler growth and the underlying mechanisms remain unclear. Here, the antler growth profile and transcriptome, plasma parameters, rumen bacteria, and metabolites (volatile fatty acids and amino acids) were determined in sika deer in a 25(OH)D supplementation group (25(OH)D, *n* = 8) and a control group (Ctrl, *n* = 8). 25(OH)D supplementation significantly increased the antler weight and growth rate. The levels of IGF-1,25(OH)D and 1,25-dihydroxyvitamin D were significantly higher in the 25(OH)D group than in the Ctrl group, while the levels of LDL-C were lower. The levels of valerate and branched-chain amino acids in the rumen fluid were significantly different between the 25(OH)D and Ctrl groups. The bacterial diversity indices were not significantly different between the two groups. However, the relative abundances of the butyrate-producing bacteria (families Lachnospiraceae and Succinivibrionaceae) and the pyruvate metabolism pathway were higher in the 25(OH)D group. The transcriptomic profile of the antler was significantly different between the 25(OH)D and Ctrl groups, with 356 up- and 668 down-regulated differentially expressed genes (DEGs) in the 25(OH)D group. The up-regulated DEGs were enriched in the proteinaceous extracellular matrix and collagen, while the down-regulated DEGs were enriched in the immune system and lipid metabolism pathways. Overall, these results provide novel insights into the effects of 25(OH)D supplementation on the host metabolism, rumen microbiota, and antler transcriptome of sika deer.

## 1. Introduction

Sika deer (*Cervus nippon*) is an important ruminant species that periodically grows velvet antlers, which is a completely regenerated organ with a fast growth rate in mammals [1]. Antlers start to grow from bony pedicles located on the head of male sika deer in the spring of each year, and antler bone growth occurs through a combination of modified endochondral ossification within each antler distal tip after cartilage formation during the growth period [2]. Furthermore, the antler is a typical example of rapid bone growth, with rates reaching 0.67 cm/day in red deer [3], 1.25 cm/day in sika deer [4], to 2.75 cm/day in elk [5]. The morphological and histological evidence demonstrates that antler growth is a manifestation of cartilage ossification, including endochondral ossification and intramembranous ossification [6]. Transcriptomic analysis has further indicated that the genes mainly involved in mesenchymal progenitor cell proliferation, chondrogenesis, and osteogenesis are responsible for the rapid growth of antlers in sika deer [7,8]. These results suggest that bone metabolism likely affects antler growth in sika deer.

Growing evidence has demonstrated that the active forms of vitamin D, 25-hydroxyvitamin D (25(OH)D) and 1,25-dihydroxyvitamin D (1,25(OH)_2_D), are essential for bone metabolism [9]. 25(OH)D directly promotes osteoclast differentiation to regulate bone resorption, while 1,25(OH)_2_D indirectly stimulates the differentiation of precursor cells into osteoclasts by inducing the production of the receptor activator nuclear factor-κB ligand [10]. The results of studies in dairy cows have also suggested that calcium absorption and bone resorption are affected by the plasma concentration of 1,25(OH)_2_D [11]. For Cervidae, it has been observed that the serum level of 1,25(OH)_2_D in white-tailed deer is associated with the antler growth rate [12]. Sempere et al. (1989) suggested that the local production of 1,25(OH)_2_D in antlers likely contributes to their bone growth in roe deer [13]. Importantly, we previously demonstrated that the efficiency of 1,25(OH)_2_D production is also associated with the growth of reindeer antlers [14]. We thus hypothesized that 25(OH)D supplementation could accelerate the antler growth profile of sika deer. However, Rodney et al. (2018) found a positive correlation in Holstein cows between 25(OH)D and insulin-like growth factor 1 (IGF-1) [15], an endocrine factor involved in regulating antler growth [16]. Vitamin D treatment increases both 25(OH)D and IGF-1 concentrations in humans [17]. Moreover, a previous study demonstrated that reserve mesenchymal stem cells (MSCs) within the antler growth center (AGC) [18] were mediated by IGF-1 [19]. Thus, there is a possibility that 25(OH)D supplementation affects the production of IGF-1 and the transcriptome of AGC to promote antler growth in sika deer.

It has also been reported that supplementation with 25(OH)D affects energy metabolism [15], promotes colostrum yield in Holstein dairy cows [20], and linearly increases the average daily gain and feed efficiency of preweaning calves [21]. It is well known that the rumen is the key location affecting feed digestion, energy metabolism, and host performance [22]. These results suggest that 25(OH)D supplementation affects rumen metabolism. Importantly, several studies have suggested an interaction between the gut microbiota and circulating vitamin D levels. Singh et al. (2020) suggested that vitamin D supplementation significantly increased gut microbial diversity and the Bacteroidetes-to-Firmicutes ratio of women [23]. Thomas et al. (2020) found that higher levels of 1,25(OH)_2_D were associated with the abundances of specific butyrate producers from the Clostridiales order [24]. Therefore, we hypothesized that 25(OH)D supplementation is related to the composition and function of the rumen microbiota in sika deer.

In this study, we aimed to investigate (i) whether antler growth and plasma parameters are associated with 25(OH)D supplementation; (ii) whether rumen microbiota features and metabolites are affected by 25(OH)D supplementation using 16S rRNA gene sequencing and gas chromatography; and (iii) how the transcriptome in the AGC responds to 25(OH)D supplementation in sika deer.

## 2. Materials and Methods

### 2.1. Animals, Experimental Design, and Diets

A total of 16 four-year-old male sika deer (mean body weight = 109.5 ± 2.1 kg) with a similar casting time of the hard antler button (around 25 April 2020) were used in this study at the research farm of Jilin Agricultural University. The sika deer were randomly assigned to two groups, were fed a total mixed ration (TMR) based on roughage and concentrate (45:55, dry matter basis, Table S1), and were randomly assigned to one of two experimental diets: a basal diet (Ctrl group, *n* = 8) or 3 mg/d 25(OH)D supplementation diet (120,000 IU per sika deer per day, 25(OH)D group, *n* = 8). The sika deer in each group were raised in an individual pen, were fed twice daily at 07:00 a.m. and 16:00 p.m., and had free access to drinking water. The experiments were conducted for 8 weeks after the removal of the hard antler button, with 1 week for adaptation followed by 7 weeks of dietary treatment. All of the animal-specific procedures were approved and authorized by the Animal Ethics Committee of Jilin Agricultural University.

### 2.2. Sample Collection and Measurement

The antlers were harvested and weighed at the end of the experiment before morning feeding. The blood samples were collected by puncture of the jugular vein of each sika deer into heparinized evacuated tubes. The AGC tissues were collected and cut into small pieces approximately 0.5 cm × 0.5 cm, and then immediately frozen in liquid nitrogen. About 200 mL rumen liquid was obtained via the stomach tube, and the first 50 mL of rumen fluid was discarded to avoid saliva contamination, and then stored in liquid nitrogen for further analysis.

The blood samples were centrifuged at 3500× *g* for 10 min at 4 °C to obtain the plasma from the heparinized evacuated tubes, and then were used to determine the concentrations of aspartate aminotransferase (AST), alanine aminotransferase (ALT), lactate dehydrogenase (LDH), total protein (TP), triglyceride (TG), total cholesterol, high-density lipoprotein cholesterol (HDL-C), low-density lipoprotein cholesterol (LDL-C), glucose, and urea nitrogen using commercial kits (Jiancheng Bioengineering Institute, Nanjing, China). The levels of plasma IGF-1, 1,25(OH)_2_D, and 25(OH)D were determined using Enzyme-linked immunosorbent assay (ELISA) kits (Jianglai Biocompany, Shanghai, China). The rumen liquid was centrifuged at 12,000× *g* for 5 min at 4 °C, the molar concentrations of volatile fatty acids (VFAs) were determined according to a previous study by gas chromatography (6890GC, Agilent Technologies, Santa Clara, CA, USA) with a flame ionization detector and a DB-FFAP column [25], and the concentrations of amino acid were measured by using ion-exchange chromatography (L8900, Hitachi Technology, Tokyo, Japan).

### 2.3. DNA and RNA Extraction and Sequencing

Microbial genomic DNA was extracted from the rumen liquid samples (five samples in each group) using a QIAamp^®^ Fast DNA Stool Mini Kit (QIAGEN, Valencia, CA, USA), according to the manufacturer’s instructions. The primers 341F (5′-CCTACGGGAGGCAGCAG-3′) and 806R (5′-GGACTACHVGGGTWTCTAAT-3′) were used to amplify the bacterial 16S rRNA gene in the V3-V4 region. Each primer pair contained the appropriate Illumina adapter sequence and an 8-bp barcode. The resulting amplicons were purified using the QIAquick PCR Purification Kit (QIAGEN, Valencia, CA, USA) and sequenced on the Illumina Novaseq 6000 to generate paired-end reads. The total RNA of the AGC tissue (five samples in each group) were isolated using Qiagen RNeasy Mini Kit (QIAGEN, Valencia, CA, USA), and checked for the RNA concentration and quality using a NanoDrop 2000 (NanoDrop, Wilmington, DE, USA). RNA samples with an integrity number (RIN) greater than 7.0 were used for the library construction. A total of 1.5 μg RNA from each sample was used to construct the RNA-Seq library using NEBNext^®^ UltraTM RNA Library Prep Kit (Illumina, San Diego, CA, USA). Each library was quantified using a Qubit 2.0 Fluorometer (Invitrogen, Carlsbad, CA, USA), and then sequenced on an Illumina HiSeq 4000 platform (150 bp paired-end sequencing).

### 2.4. Bioinformatics Analysis

For the 16S rRNA sequences, the paired-end sequences were first assembled into contigs using FLASH [26]. The obtained contigs were then processed using QIIME 1.9.1 [27]. The sequences were clustered into operational taxonomic units (OTUs) with 97% sequence identity using UPARSE [28], and the potential chimeras were identified and removed using UCHIME [29]. The representative sequences of each OTU were assigned against the SILVA database (v138) [30]. Singletons were removed and each sample was sub-sampled with a minimum number (37,535) to reduce the effects of the sequencing depth. Alpha-diversity indices were also calculated using QIIME 1.9.1. Principal coordinates analysis (PCoA) based on Bray–Curtis dissimilarity matrices were performed to reveal the differences in rumen bacterial communities between the two groups. PERMANOVA was performed to indicate group difference, and the *p* values were determined based on 999 permutations. The phylogenetic investigation of communities by reconstruction of the unobserved states (PICRUSt) was applied to predict the functional profiles of the rumen microbiota resulting from reference-based OTU picking against the Greengenes database [31]. The predicted genes were then summarized according to the Kyoto Encyclopedia of Genes and Genomes (KEGG) pathways.

For the transcriptome analysis, Trimmomatic was used to remove the low quality and adapter sequence [32]. After that, HISAT2 was used to align the remaining clean reads to the reference genome of the sika deer [33]. StringTie was applied to estimate the expression of each gene transcript by fragments per kilobase of transcript per million fragments mapped (FPKM) [34]. The differentially expressed genes (DEGs) were determined using the edgeR package [35] by |log_2_(FC)| > 0.5 and with a false discovery rate (FDR < 0.05) based on the Benjamini and Hochberg correction. The principal component analysis (PCA) was applied to reveal the gene expression of AGC tissues between the two groups. The significantly enriched gene ontology (GO) term and KEGG pathways of DEGs were performed by KOBAS using a false discovery rate (FDR < 0.05) based on Benjamini and Hochberg correction [36]. The gene-concept networks for DEGs were performed using clusterProfiler [37].

### 2.5. Statistical Analysis

The Wilcoxon rank sum (WRS) test was used to determine the statistical significance of the antler weight, antler growth, plasma parameter levels, volatile fatty acids (VFAs) and amino acid concentrations in the rumen fluid, alpha diversity indices, the relative abundance of bacteria according to taxonomy, and the enrichment of the KEGG pathways between the Ctrl and 25(OH)D groups. The *p* values of the WRS tests were corrected using the false discovery rate of the Benjamini-Hochberg method. Linear regression analysis was used to estimate the association between the plasma 25(OH)D and IGF-1 concentrations. All *p* values ≤ 0.05 were considered to indicate statistical significance.

## 3. Results

### 3.1. Increased Antler Production and Altered Plasma Parameters

**Figure 1:**
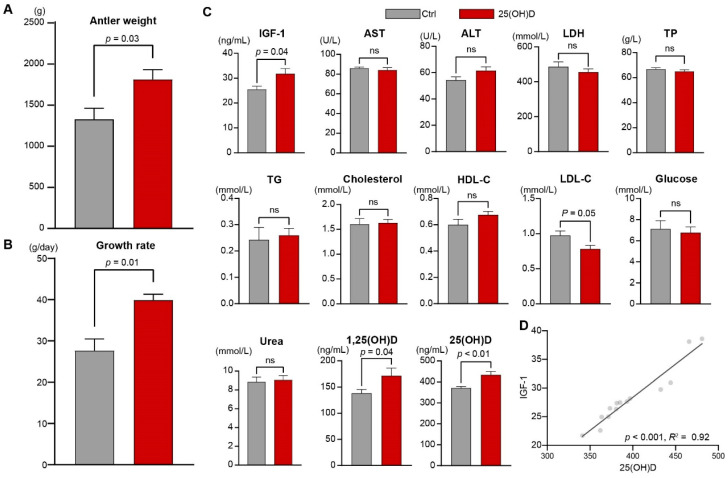
Changes in antler production performances and host metabolism with 25(OH)D supplementation. Comparison of the (**A**) antler weight, (**B**) growth rate of the antler, and (**C**) plasma metabolites between the Ctrl and 25(OH)D groups. ns means no significant differences. *p* value indicates the statistical significance of the differences, which were determined using WRS test based on the Benjamini–Hochberg correction. (**D**) The correlation between plasma 25(OH)D and IGF-1 concentrations. *p* value indicates the statistical significance of the differences, which were determined using liner regression analysis. The R^2^ value indicates the coefficient of determination. IGF-1: Insulin Growth Factor-1, AST: Aminotransferase, ALT: Alanine Aminotransferase, LDH: Lactate Dehydrogenase, TP: Total Protein, TG: Triglyceride, HDL-C: High-Density Lipoprotein Cholesterol, LDL-C: Low-Density Lipoprotein Cholesterol, 1,25(OH)D: 1,25-Dihydroxyvitamin D, 25(OH)D: 25-Hydroxyvitamin D.

We first evaluated the production performance of the antlers and found that the final weight (*p* = 0.03, Figure 1A) and daily growth weight of the antlers (*p* = 0.01, Figure 1B) in the 25(OH)D group were significantly greater than those in the Ctrl group. Further examination of the plasma parameters showed that the levels of IGF-1 (*p* = 0.04), 25(OH)D (*p* < 0.01), and 1,25(OH)_2_D (*p* = 0.04) were significantly higher in the 25(OH)D group than in the Ctrl group. The level of LDL-C in the 25(OH)D group (*p* = 0.05) was lower than that in the Ctrl group (Figure 1C). Then, we found an apparently positive association between the plasma 25(OH)D and IGF-1 concentrations (*p* < 0.001, *R*^2^ = 0.92) based on linear regression analysis (Figure 1D).

### 3.2. Variations in VFA and Amino Acid Profiles in the Rumen

**Figure 2:**
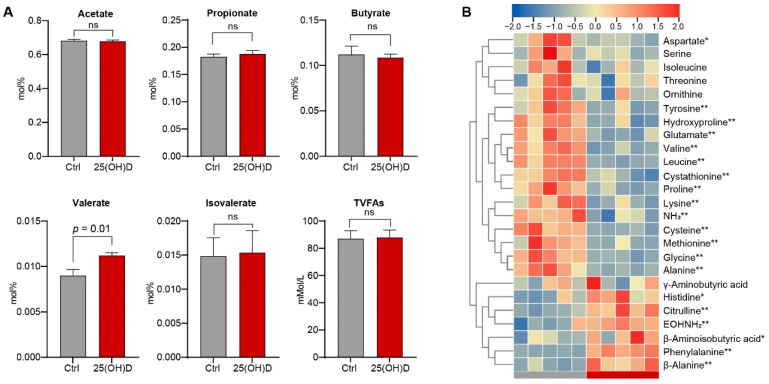
The changed production of VFAs and amino acids in rumen liquid between the Ctrl and 25(OH)D groups. (**A**) Comparison of VFAs in the rumen liquid between the Ctrl and 25(OH)D groups. ns means no significant differences. (**B**) Heatmap showing the concentrations of amino acids in rumen liquid between the Ctrl and 25(OH)D groups. Square colors indicate the amino acids concentration from low (blue) to high (red). Samples from the Ctrl group and 25(OH)D group were indicated by grey and red bars at bottom of heatmap. *p* value indicates the statistical significance of the differences, and were determined using WRS test based on the Benjamini–Hochberg correction. ** and * indicate *p* < 0.01 and *p* < 0.05, respectively. TVFAs: Total Volatile Fatty Acids.

The results showed that the concentration of total VFAs (*p* = 0.82) and the molar proportions of acetate (*p* = 0.77), propionate (*p* = 0.53), butyrate (*p* = 0.74), and isovalerate (*p* = 0.91) were not significantly different between the Ctrl and 25(OH)D groups (Figure 2A). However, the molar proportion of valerate in the 25(OH)D group was significantly greater than that in the Ctrl group (*p* = 0.01), indicating the alteration of the metabolism of branched-chain amino acids (BCAAs) in the rumen. Thus, we further compared the amino acid concentrations between the two groups. The results showed that a total of 11 amino acids, including aspartate, glutamate, glycine, alanine, valine, cysteine, methionine, leucine, tyrosine, lysine, and proline, as well as their related metabolites (cystathionine, ammonia, and hydroxyproline), were significantly enriched in the Ctrl group compared with those in the 25(OH)D group (*p* < 0.05); however, the concentrations of citrulline, phenylalanine, β-alanine, histidine, β-aminoisobutyric acid, and EOHNH_2_ were lower (*p* < 0.05, Figure 2B).

### 3.3. 25(OH)D Supplementation Alters the Rumen Bacterial Composition

**Figure 3:**
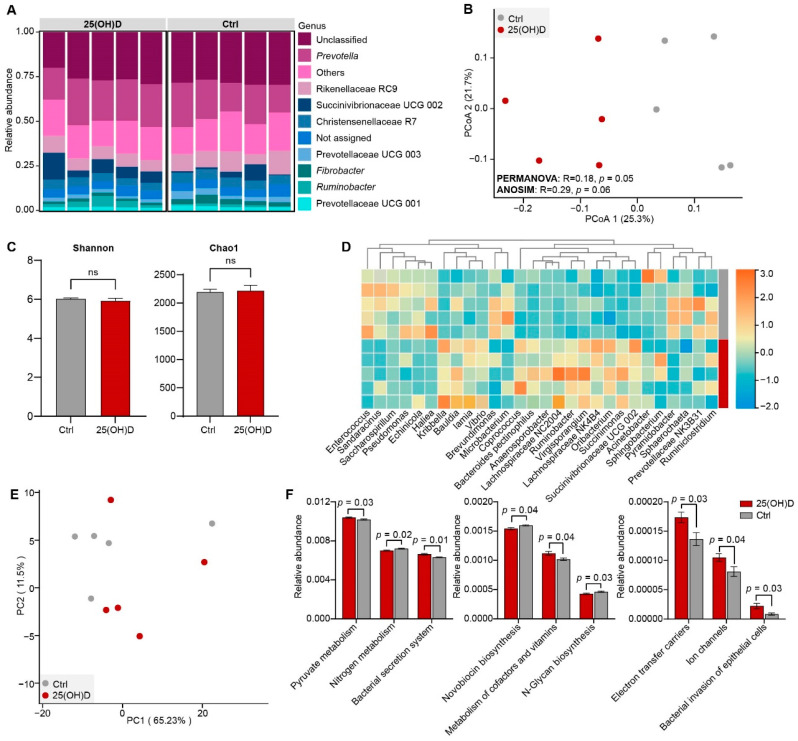
Rumen bacterial community and the predicted functional profiles. (**A**) Bacterial composition in the rumen fluid of sika deer between the Ctrl and 25(OH)D groups at the genus level. (**B**) Comparison of the alpha diversity indices in the rumen fluid of sika deer between the Ctrl and 25(OH)D groups. ns means no significant differences. (**C**) PCoA results showing the separation of the rumen bacteria between Ctrl and 25(OH)D groups at the OTU level based on the Bray–Curits dissimilarity matrix. (**D**) Heatmap showing the significantly changed bacteria genera in the rumen liquid between the Ctrl and 25(OH)D groups. Square colors indicate the normalized relative abundance of each genera from the minimum (blue) to maximum (orange). Rumen liquid samples from the Ctrl group and 25(OH)D group were indicated by grey and red bars on the right side of the heatmap, respectively. (**E**) PCA revealing the difference in the bacterial functional profiles at KEGG level 3 (relative abundance) based on the Euclidean metric. (**F**) Histograms showing the significantly changed pathways at KEGG level 3.

Then, we examined the rumen bacterial community based on the 16S rRNA sequences. A total of 440,260 high-quality reads were obtained, with an average of 44,026 sequences for each sample (37,535 to 51,506). After rarefication to the minimum number, we obtained a total of 3464 OTUs based on 97% sequence identity. Based on these OTUs, we identified a total of 48 phyla in the rumen liquid from the two groups (Figure S1A), dominated by the phyla Bacteroidota, Firmicutes, and Proteobacteria (Figure S1B). At the genus level, the identified OTUs were further classified into 283 genera (Figure 3A). In the Ctrl group, *Prevotella* (20.12%) was the most dominant bacteria, followed by bacteria belonging to Rikenellaceae RC9 (9.88%), Christensenellaceae R7 (5.35%), Prevotellaceae UCG 003 (3.20%), and *Fibrobacter* (3.04%), accounting for ~45.0% of the overall bacterial composition. In the 25(OH)D group, the genus *Prevotella* (22.76%) was predominant, followed by bacteria belonging to Succinivibrionaceae UCG 002 (7.80%), Rikenellaceae RC9 (7.56%), Christensenellaceae R7 (4.76%), and *Ruminobacter* (3.11%), accounting for 46.0% of the bacterial composition.

We further revealed the difference in bacterial community composition between the Ctrl and the 25(OH)D groups. The PCoA results showed that the bacterial community composition in the rumen of the 25(OH)D group was distinct from that in the Ctrl group, based on Bray-Curtis dissimilarity matrices (Figure 3B), explaining at least 47.0% of the variation. The results of PERMANOVA also indicated a significant difference between the Ctrl and the 25(OH)D groups. However, the Shannon and Chao1 indices were not significantly different between the Ctrl and the 25(OH)D groups (Figure 3C).

Moreover, the relative abundances of *Anaerosporobacter*, *Bauldia*, *Coprococcus*, *Iamia*, *Kribbella*, *Oribacterium*, *Ruminobacter*, *Succinimonas*, *Vibrio*, and *Virgisporangium*, bacteria belonging to Lachnospiraceae NC2004, Lachnospiraceae NK4B4, and Succinivibrionaceae UCG 002, were significantly higher in the 25(OH)D group than in the Ctrl group (*p* < 0.05, Figure 3D). In contrast, the relative abundances of *Acinetobacter*, *Enterococcus*, *Pyramidobacter*, *Ruminiclostridium*, *Saccharospirillum*, *Sphaerochaeta*, *Sphingobacterium*, and bacteria, belonging to Prevotellaceae NK3B31 were significantly higher in the Ctrl group than in the 25(OH)D group (*p* < 0.05, Figure 3D).

We then applied PICRUSt to predict the potential functions of the rumen bacteria and compared the difference between the two groups. The PCA results showed that the bacterial metabolic pathways at the KEGG level 3 in the 25(OH)D group separated from that in the Ctrl group (Figure 3E). Moreover, the relative abundances of the pyruvate metabolism, the bacterial secretion system, electron transfer carriers, ion channels, and bacterial invasion of the epithelial cell were increased in the 25(OH)D group compared with those in the Ctrl group (*p* < 0.05, Figure 3F). However, the pathways of nitrogen metabolism, N-Glycan biosynthesis, and novobiocin biosynthesis were significantly decreased in the 25(OH)D group (*p* < 0.05, Figure 3F).

### 3.4. 25(OH)D Supplementation Leads to Extensive Transcriptome Changes in AGC Tissue

**Figure 4:**
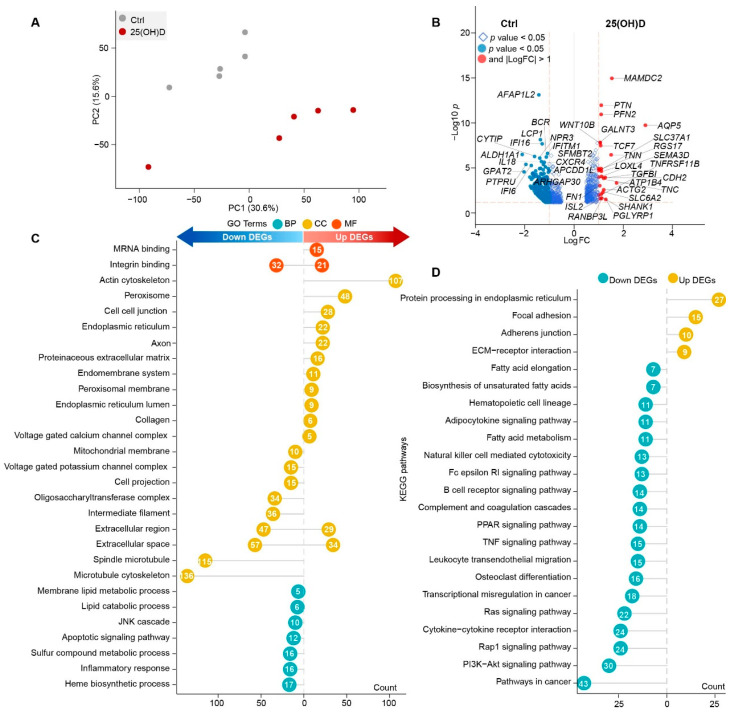
25(OH)D supplementation affects the gene expression of the AGC tissue. (**A**) PCA analysis of the gene expression in the AGC tissue of sika deer between the Ctrl and 25(OH)D groups. (**B**) Volcano plots show up- and down-DEGs in the AGC tissue between two groups. Red and blue dots indicate up- and down-regulated DEGs (*p* < 0.05 and |log_2_ (FC)| > 1) in the 25(OH)D group compared to Ctrl group. FC = fold change. The enriched GO terms (**C**) and KEGG pathways (**D**) of the up- and down-regulated DEGs in the 25(OH)D group in comparison with the Ctrl group. Numbers in circles indicate the number of DEGs.

We next examined whether the genes were differentially expressed in the AGC tissue after 25(OH)D supplementation. RNA sequencing was performed, and we confirmed that dietary 25(OH)D supplementation affected the gene expression of the AGC, as indicated by the PCA results, explaining approximately 46% of the total variation (Figure 4A). We identified 1024 DEGs in the AGC of sika deer in the 25(OH)D group compared with the Ctrl group (356 up-regulated and 668 down-regulated, Table S2), indicating that many genes within AGC are responsive to 25(OH)D supplementation. The up-regulated DEGs included *MAMDC2*, *PTN*, *FN1*, *TNC*, *TNN*, *TGFBI*, *CDH2*, *TNFRSF11B*, *TCF7*, and *WINT10B*, while the down-regulated DEGs included *AFAP1L2*, *LCP1*, *IFI16*, *BCR*, *CYTIP*, *ALDH1A1*, *IL18*, *GPAT2*, *PTPRU*, *IF16*, *NPR3*, *IFITM1*, *SFMBT2*, *CXCR4*, *APCDD1L*, and *ARHGAP30* (Figure 4B). These up-regulated DEGs were enriched in 13 Gene Ontology (GO) cell component categories, including actin cytoskeleton, cell-cell junction, peroxisome, axon, proteinaceous extracellular matrix, and collagen. They were also enriched in two GO molecular function categories, namely, mRNA binding and integrin binding (Figure 4C). For the 668 down-regulated DEGs, according to the GO analysis of the cellular components, these DEGs were significantly enriched for microtubule cytoskeleton, spindle microtubule, extracellular space, extracellular region, and intermediate filament. They were also enriched for the biological function GO terms involved in the heme biosynthetic process, inflammatory response, lipid catabolic process, and membrane lipid metabolic process (Figure 4C).

According to the KEGG analysis, the up-regulated DEGs were enriched in only a few pathways, including protein processing in the endoplasmic reticulum, focal adhesion, adherens junction, and ECM–receptor interaction (Figure 4D). In contrast, the down-regulated DEGs were significantly enriched in the pathways of the immune response (cytokine-cytokine receptor interaction, leukocyte transendothelial migration, B cell receptor signaling, and natural killer cell mediated cytotoxicity), lipid metabolism (fatty acid elongation, biosynthesis of unsaturated fatty acids, fatty acid metabolism, and adipocytokine signaling), and signaling pathways (PI3K−Akt signaling, Rap1 signaling, Ras signaling, TNF signaling, and peroxisome proliferator-activated receptor (PPAR) signaling pathways, Figure 4D).

**Figure 5:**
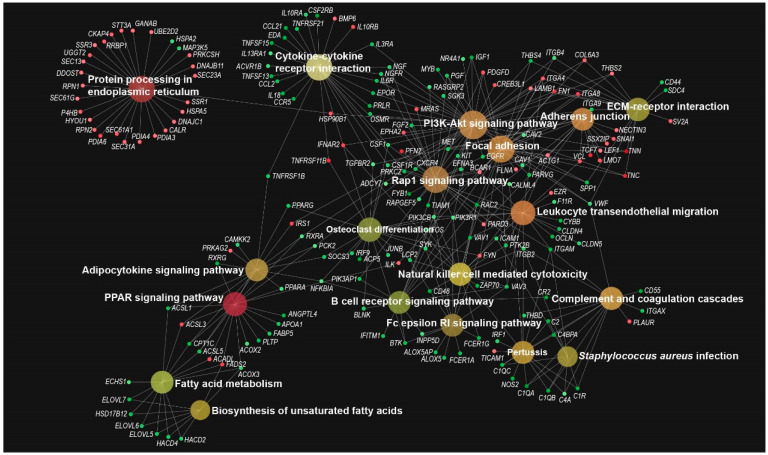
Identification of the transcriptional enrichment networks. The enrichment pathway was calculated using the gene set enrichment analysis. Each significantly enriched KEGG pathway is represented as a circle. Circle colors indicate the significance of the enriched KEGG pathways from minimum (red) to maximum (yellow). The circle size indicates the number of DEGs. Gene sets with overlapping genes are connected with an edge. Red and green nodes indicate up- and down-regulated DEGs in the 25(OH)D group compared with the Ctrl group. The network is displayed as the Bipartite layout.

To further interpret the enrichment of the KEGG pathways, we conducted an interactive enrichment network analysis based on the up- and down-regulated DEGs. The results showed that the pathways of the biosynthesis of unsaturated fatty acids, adipocytokine signaling pathway, and PPAR signaling pathway were closely associated, and were connected by genes such as *ELOVL5*, *ELOVL6*, *ELOVL7*, *PPARA*, *PPARG*, *ACSL1*, *ACSL3*, *ACSL5*, *ACADL*, and *FADS2*. Moreover, the cytokine-cytokine receptor interaction, osteoclast differentiation, B cell receptor signaling pathway, PI3K-Akt signaling pathway, natural killer cell mediated cytotoxicity, and Rap1 signaling pathway, were also closely connected, involving genes such as *NGF*, *NGFR*, *IL6R*, *EPOR*, *PRLR*, *IL3RA*, *IFNAR2*, *TNFRSF11B*, *IFNAR2*, *TGFBR2*, *CSF1*, *LCP2*, *SYK*, and *BLNK* (Figure 5). Moreover, the enrichment network analysis also showed that the GO biological process categories, including RNA splicing via transesterification reactions, inorganic anion transport, the apoptotic signaling pathway, regulation of binding, and inflammatory response, were closely associated, while the GO molecular function categories, including integrin binding and mRNA binding, were also identified (Figure S2).

## 4. Discussion

The results of this study indicate that 25(OH)D supplementation significantly increased the antler final weight and growth rate and 25(OH)D and 1,25(OH)_2_D levels in the plasma. Consistent with our observation, 25(OH)D supplementation can promote 1,25(OH)_2_D synthesis in weaning calves [38]. Previous studies have also demonstrated that the 1,25(OH)_2_D concentration was significantly increased during antler growth in white-tailed deer [12] and fallow deer [39]. These results thus confirmed the regulatory role of 25(OH)D and 1,25(OH)_2_D on antler growth. We also found a positive correlation between the plasma concentrations of 25(OH)D and IGF-1, an important antler-stimulating hormone [16]. Consistently, Bogazzi et al. (2011) also observed that IGF-1 concentrations were significantly correlated with 25(OH)D levels in humans [40]. Moreover, a significant decrease was observed in the plasma levels of LDL-C upon 25(OH)D supplementation. It has been reported that a reduced IGF-1 concentration impairs the expression of the genes encoding the low-density lipoprotein receptors that are involved in lipid catabolism [41]. These findings suggest a possible interaction among 25(OH)D, IGF-1 and lipid metabolism during antler growth.

We further examined whether 25(OH)D supplementation affected rumen metabolism. The results showed that the molar proportion of valerate was significantly increased, and the concentrations of amino acids significantly differed in the 25(OH)D group. Valerate is an end-product of proline, lysine, and methionine degradation [42]. Accordingly, the proline, lysine, and methionine concentrations were significantly decreased in the rumen of the 25(OH)D group. Moreover, the concentration of leucine decreased, whereas that of isovalerate increased. Isovalerate is a product of BCAAs, such as leucine [43]. It has been observed that vitamin D_3_ supplementation significantly lowers the circulating levels of BCAAs in type two diabetes mellitus [44]. These results suggest that 25(OH)D supplementation may affect BCAAs metabolism in the rumen of sika deer.

We further investigated how the rumen bacterial community was affected by 25(OH)D supplementation, due to its key role in rumen metabolism, and confirmed the dominance of *Prevotella* in the rumen of sika deer [45]. However, the rumen bacterial composition was significantly different between the 25(OH)D group and the Ctrl group, consistent with the observations in healthy humans, which indicated that the Bacteroidetes-to-Firmicutes ratio increased with vitamin D supplementation [23], and that the abundances of specific taxonomies (Clostridia order) were associated with the level of 1,25(OH)_2_D [24]. The relative abundance of members belonging to the Succinivibrionaceae family (Succinivibrionaceae UCG 002 and *Ruminobacter*) and Lachnospiraceae family (*Coprococcus*, *Oribacterium*, Lachnospiraceae NC2004, *Anaerosporobacter,* and Lachnospiraceae NK4B4) significantly increased in the 25(OH)D group. Members of the Succinivibrionaceae family mainly capture hydrogen to produce succinate, thereby promoting the production of VFAs [46], and are associated with rumen energy metabolism in sika deer [25]. Our findings support previous observations of a dynamic interplay between active vitamin D metabolites and the Lachnospiraceae family [24]. Members within the Lachnospiraceae family are important butyrate-producing bacteria [47]. We also found that the relative abundance of pyruvate metabolism was increased in the 25(OH)D group. These results suggest that butyrate producers were affected by 25(OH)D supplementation in the rumen of sika deer.

We then examined whether and how the gene expression profiles in AGC responded to 25(OH)D supplementation. We identified 356 up-regulated DEGs in the 25(OH)D group in comparison with those in the Ctrl group, including *FN1*, *TNC*, *TNN*, *TGFBI*, *CDH2*, *TNFRSF11B*, *TCF7*, *WINT10B,* and *PTN*. *FN1* encodes the extracellular matrix (ECM) protein and is a vitamin D target [48], whose expression linearly increased during the antler growth period [49], indicating a direct role of 25(OH)D in antler growth. The overexpression of *TNFRSF11B*, encoding an osteoclastogenesis inhibitory factor, resulted in a strong upregulation of cartilage extracellular matrix components [50]. *CDH2*, *WNT10B,* and *PTN* are highly expressed genes in the antler. The former mediates cell-cell interactions and enhances MSC aggregation, resulting in chondrogenic differentiation [51], while the latter two genes regulate the differentiation of antler chondrocytes [7]. *TCF7* is considered a regulator of early osteoblast differentiation of MSCs [52]; *TGFBI* is a core gene regulating antler growth and ossification [53]; and *PTN* has been identified as a critical factor promoting cartilage regeneration, angiogenesis, and chondrogenesis [54]. In addition, previous findings revealed that 1,25(OH)_2_D regulated cell-to-cell communication by post-transcriptionally altering the expression of the genes involved in ECM organization and receptor interactions [55]. Interestingly, these up-regulated DEGs were enriched in the pathways of the ECM−receptor interaction and protein processing in the endoplasmic reticulum. The statistically enriched biological cellular component terms were proteinaceous extracellular matrix and collagen. In this context, it is relevant that the chondrocyte differentiation and osteoblast ossification pathways were up-regulated by 25(OH)D supplementation, leading to increased antler growth.

Notably, following 25(OH)D supplementation, the majority of changes (65.2%) in the gene expression reflected downregulation, and down-regulated genes were enriched in different pathways of the immune system, such as the B cell receptor signaling pathway, complement and coagulation cascades, and cytokine−cytokine receptor interaction. The immunomodulatory effects of vitamin D on both innate and adaptive immunity are well documented [56], indicating that the immune response in antlers was affected by 25(OH)D supplementation. It has been reported that 1,25(OH)_2_D stimulates IL-10 release in dendritic cells, towards a less mature and more tolerogenic phenotype, resulting in a reduction in the anti-inflammatory activity of macrophages (i.e., IL-6 and TNF-α) [56]. In our study, 25(OH)D supplementation selectively down-regulated the expression of TNF superfamily members 1B, 13, 15, and 21 and up-regulated the IL-10 receptor in AGC. Thus, our observation indicated that a physiological role of 25(OH)D supplementation is to potentially shift the immune system to a more tolerogenic status. Moreover, the *IL18* and *IFI16* expression levels were significantly down-regulated in the 25(OH)D group. This finding is consistent with previous observations in mice, that 1,25(OH)_2_D markedly suppressed the expression of *IL18* in primary keratinocytes and modulated the cutaneous inflammatory reactions [57]. The DNA sensor IFI16 is essential for DNA-driven innate immune responses in keratinocytes, is up-regulated in psoriatic skin lesions, and is localized to the cytoplasm in a subpopulation of cells [58]. Keratinocytes are involved in protecting the body from infections and environmental challenges. These results further indicate the downregulation of the immune response in the AGC by 25(OH)D supplementation and a possible balance between the immune response and antler growth.

Another major finding of the transcriptome analysis was the enrichment of down-regulated DEGs in lipid metabolism pathways (i.e., *ELOVL5*, *ELOVL6*, *ELOVL7*, *PPARA,* and *PPARG*), including fatty acid elongation, biosynthesis of unsaturated fatty acids, the adipocytokine signaling pathway, and the PPAR signaling pathway. Moreover, the enriched biological process terms were the membrane lipid metabolic process and lipid catabolic process. This result suggests that 25(OH)D supplementation might result in the regulation of lipid metabolic regulatory pathways via target genes. Seven distinct elongation of very-long-chain fatty acid (FA) enzymes (ELOVL1–ELOVL7) play important roles in the elongation of medium to long-chain FAs into very-long-chain FAs [59]. In addition, the loss of function of the vitamin D–vitamin D receptor (VD-VDR) system in mice resulted in increased levels of very-long-chain FAs (C18–C24) in the white adipose tissue [60]. The decreased expression level of *ELOVL*s indicates a potential inhibitory effect of 25(OH)D supplementation on the synthesis of long-chain FAs in antlers. PPARα and PPARγ are key regulators of lipid homeostasis and are activated by a structurally diverse group of compounds, including long-chain arachidonic acid [61], which is mediated by the enzyme ELOVL5 [62]. However, the VD-VDR system has also shown an anti-PPARG activity, as inhibiting its expression leads to adipogenesis in the adipocyte cells [63], suggesting that long-chain FAs might be involved in the PPAR signaling pathway. This hypothesis is also supported by the connection of the metabolic pathway by acyl-CoA synthetases long-chain (ACSLs), the key enzymes converting nonpolar hydrophobic FA substrates into acyl-CoAs. Regarding increased levels of 25(OH)D and 1,25(OH)_2_D, the results of the present study reveal the possibility of the interaction of vitamin D, long-chain FAs, and the PPAR signaling pathway in the regulation of metabolic homeostasis and antler growth.

## 5. Conclusions

Our results suggest that 25(OH)D supplementation (i) increases the antler weight and growth rate of sika deer; (ii) alters the rumen bacterial community composition and metabolic profiles, particularly regarding butyrate producers and the amino acid concentrations; and (iii) affects the genes expression of the immune response and lipid metabolism in antlers. Future studies are needed to elucidate the relationship between FA composition and antler growth. Taken together, these observations provide a better understanding of the effects of 25(OH)D supplementation on sika deer.

## Data Availability

Sequence files associated with each sample have been submitted to the NCBI Sequence Read Archive. This data can be found here: https://www.ncbi.nlm.nih.gov/sra. (accessed on 8 October 2022. PRJNA888231 and PRJNA888245)

## Supplementary Materials

The following supporting information can be downloaded at: https://www.mdpi.com/article/10.3390/ani12243497/s1. Table S1. Dietary composition and nutrient levels. Table S2. The DEGs based on |Log_2_(FC)| > 0.5 and adjust *p* < 0.05. Figure S1. Rumen bacterial composition and core microbiome profilers. (A) Bacterial composition in the rumen fluid of sika deer between Ctrl and 25(OH)D groups at the phylum level. (B) Identification of core microbiome in the rumen fluid of sika deer during antler growth period. Figure S2. Gene-concept networks based on enrichment GO terms between Ctrl and 25(OH)D groups. The enrichment biology process (A) and molecular function (B) were calculated using the gene set enrichment analysis. Each significantly enriched GO term is represented as a circle. Circle colors indicate the significance of the enriched terms from minimum (red) to maximum (yellow). Circle size indicates the number of DEGs. Gene sets with overlapping genes are connected with an edge. Red and green nodes indicate up- and down-regulated DEGs in 25(OH)D group compared with the Ctrl group. The network is displayed as the Bipartite layout.
