# Supplementary material for: Integrated Transcriptome and Microbiota Reveal the Regulatory Effect of 25-Hydroxyvitamin D Supplementation in Antler Growth of Sika Deer
Source: Animals (Basel). 2022 Dec 11;12(24):3497. doi: 10.3390/ani12243497 (PMC9774409; doi:10.3390/ani12243497)
Supplement: Supplementary file 1 [file animals-12-03497-s001.zip › Supplementary Materials 1_Table S1.pdf]

Table S1. Ingredients and chemical compositions of the experimental diets used in this study

Ingredient, (g/100 g) DM		Nutrient composition, % DM	
Corn silage	30.0	Crude protein	16.74
Alfalfa	15.0	Neutral detergent fiber	58.94
Corn grain	15.0	Acid detergent fiber	32.54
Soybean meal	20.0	ME, MJ/kg	10.78
DDGS	5.5		
Corn germ	8.0		
Corn fiber	5.0		
NaCl	0.5		
Premix[1]	1.0		
Total	100.00		

DM = Dry Matter, DDGS = Distillers Dried Grains with Solubles, ME = Metabolic Energy

1. Formulated to provide (per kg of DM): 150 g of salt, 200 g of $NaHCO_3$, 75 g of Ca, 20 g of P, 600 mg of Mn, 680 mg of Fe, 960 mg of Zn, 300 mg of Cu, 140,000 IU of vitamin A, 2,000 IU of vitamin D_3, 700 IU of vitamin E.
