# Supplementary figures and images for: Integrated Transcriptome and Microbiota Reveal the Regulatory Effect of 25-Hydroxyvitamin D Supplementation in Antler Growth of Sika Deer
Source: Animals (Basel). 2022 Dec 11;12(24):3497. doi: 10.3390/ani12243497 (PMC9774409; doi:10.3390/ani12243497)

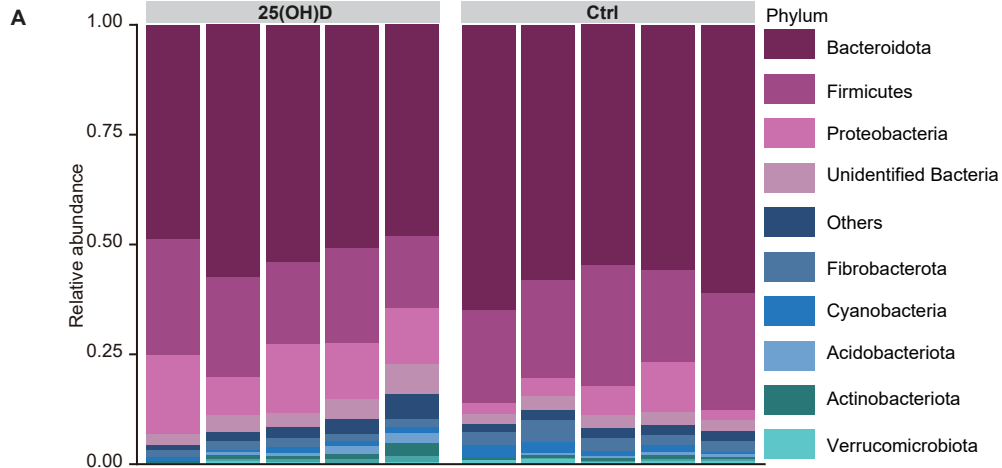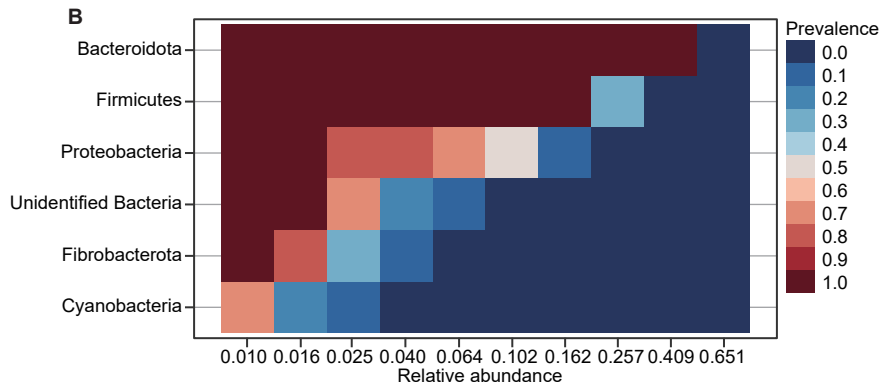

Supplement: Supplementary file 1 [file animals-12-03497-s001.zip › Supplementary Materials 2_Figure S1.pdf]

**A:BP**

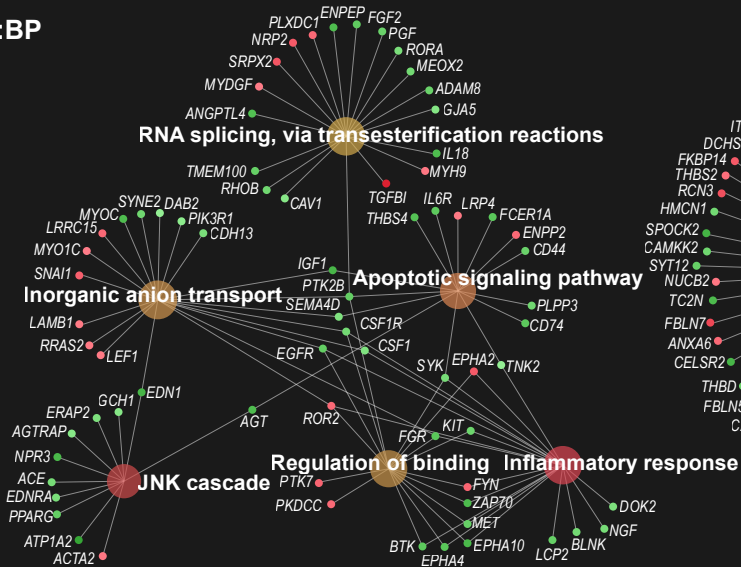

**B:MF**

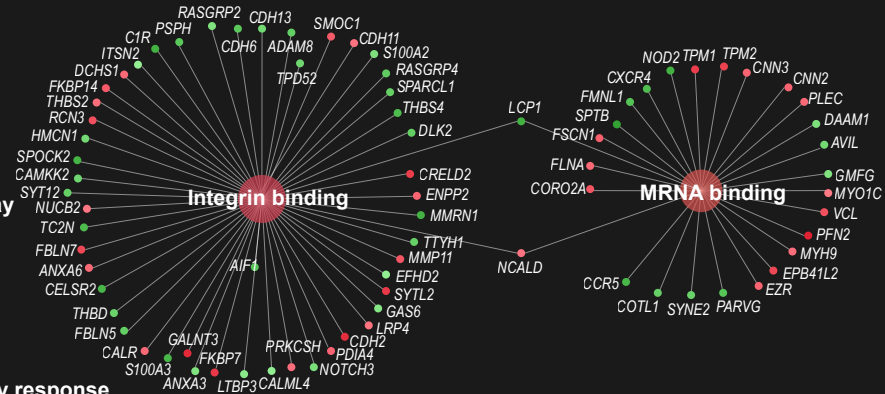

Supplement: Supplementary file 1 [file animals-12-03497-s001.zip › Supplementary Materials 3_Figure S2.pdf]
